# Correlation of bone density measured on CT chest with the severity of COVID-19 infection: A retrospective study

**DOI:** 10.1371/journal.pone.0286395

**Published:** 2023-06-08

**Authors:** Noha Bakhsh, Mai Banjar, Mukhtiar Baig

**Affiliations:** 1 Faculty of Medicine in Rabigh, Department of Medicine, Division of Radiology, King Abdulaziz University, Jeddah, Saudi Arabia; 2 Department of Medical Imaging, King Abdullah Medical Complex, Jeddah, Saudi Arabia; 3 Faculty of Medicine in Rabigh, Department of Clinical Biochemistry, King Abdulaziz University, Jeddah, Saudi Arabia; Acibadem Maslak Hospital: Acibadem Maslak Hastanesi, TURKEY

## Abstract

**Purpose:**

This retrospective study investigated the correlation between bone mineral density (BMD) and COVID-19 severity among COVID-19 patients who underwent chest computed tomography (CT) scans.

**Methods:**

This study was carried out at the King Abdullah Medical Complex in Jeddah, Saudi Arabia, one of the largest COVID-19 centers in the western province. All adult COVID-19 patients who had a chest CT between January 2020 and April 2022 were included in the study. The pulmonary severity scores (PSS) and vertebral BMD measurements were obtained from the patient’s CT chest. Data from the patients’ electronic records were collected.

**Results:**

The average patient age was 56.4 years, and most (73.5%) patients were men. Diabetes (n = 66, 48.5%), hypertension (n = 56, 41.2%), and coronary artery disease (n = 17, 12.5%) were the most prevalent comorbidities. Approximately two-thirds of hospitalized patients required ICU admission (64%), and one-third died (30%). The average length of stay in the hospital was 28.4 days. The mean CT pneumonia severity score (PSS) was 10.6 at the time of admission. Patients with lower vertebral BMD (< = 100) numbered 12 (8.8%), while those with higher vertebral BMD (>100) numbered 124 (91.2%). Only 46 out of the total survived patients (n = 95) were admitted to the ICU versus all deceased (P<0.01). The logistic regression analysis revealed that an elevated PSS upon admission resulted in a reduced chance of survival. Age, gender, and BMD did not predict survival chances.

**Conclusion:**

The BMD had no prognostic advantage, and the PSS was the significant factor that could have predicted the outcome.

## Introduction

COVID-19 is caused by severe acute respiratory syndrome coronavirus 2 (SARS-CoV-2). COVID-19 was first identified in Wuhan, China, and on March 11, 2020, it was declared a pandemic by the World Health Organization (WHO) [1,2]. Although most countries have achieved sufficient control of the pandemic and the world is now in the post-pandemic era, clinical observations and record analyses are ongoing [3].

COVID-19 is primarily a respiratory disorder. However, symptoms and effects can negatively impact the hematological, cardiac, endocrine, metabolic, neurological, gastrointestinal, and musculoskeletal systems [4,5]. Age and several comorbidities have been identified as risk factors for severe COVID. These comorbidities include but are not limited to diabetes mellitus, hypertension, cardiovascular diseases, renal diseases, and obesity [6].

Due to the highly contagious nature, early identification, isolation, and treatment are essential. Reverse transcriptase polymerase chain reaction (RT-PCR) is the gold standard for diagnosing COVID-19. However, results can be falsely negative due to low viral load initially. Computed tomography (CT) scan of the chest has added to the patient’s diagnosis, treatment, and follow-up.

Pulmonary severity score (PSS)was used to indicate the severity of the lung findings [7–10]. The researchers highlighted extrapulmonary findings on CT chest as an added benefit. Pre-existing comorbidities are linked to findings like BMD and liver density, which can impact the prognosis of COVID disease [11,12].

Osteoporosis is prevalent in Saudi Arabia [13]. Bone health and osteoporosis in the era of COVID have attracted much attention [5,14]. In addition, an association between smoking and chronic lung disease with decreased BMD was studied [15,16]. There is considerable disagreement among researchers about whether low BMD is an independent prognostic factor for poor disease outcomes [11,14,17].

To the best of our knowledge, this is the first study in Saudi Arabia to assess the relationship between BMD and the severity of COVID-19 respiratory disease. This retrospective study aimed to investigate the correlation between decreased BMD and COVID-19 severity in COVID-19 patients who underwent chest CT scans.

## Materials and methods

The institutional review board at the Ministry of Health in Saudi Arabia approved this study (registration numbers KACST, KSA: H-02-J-002), and the written consent form was waived due to the study’s retrospective and observational nature.

### Study population

**Fig 1:**
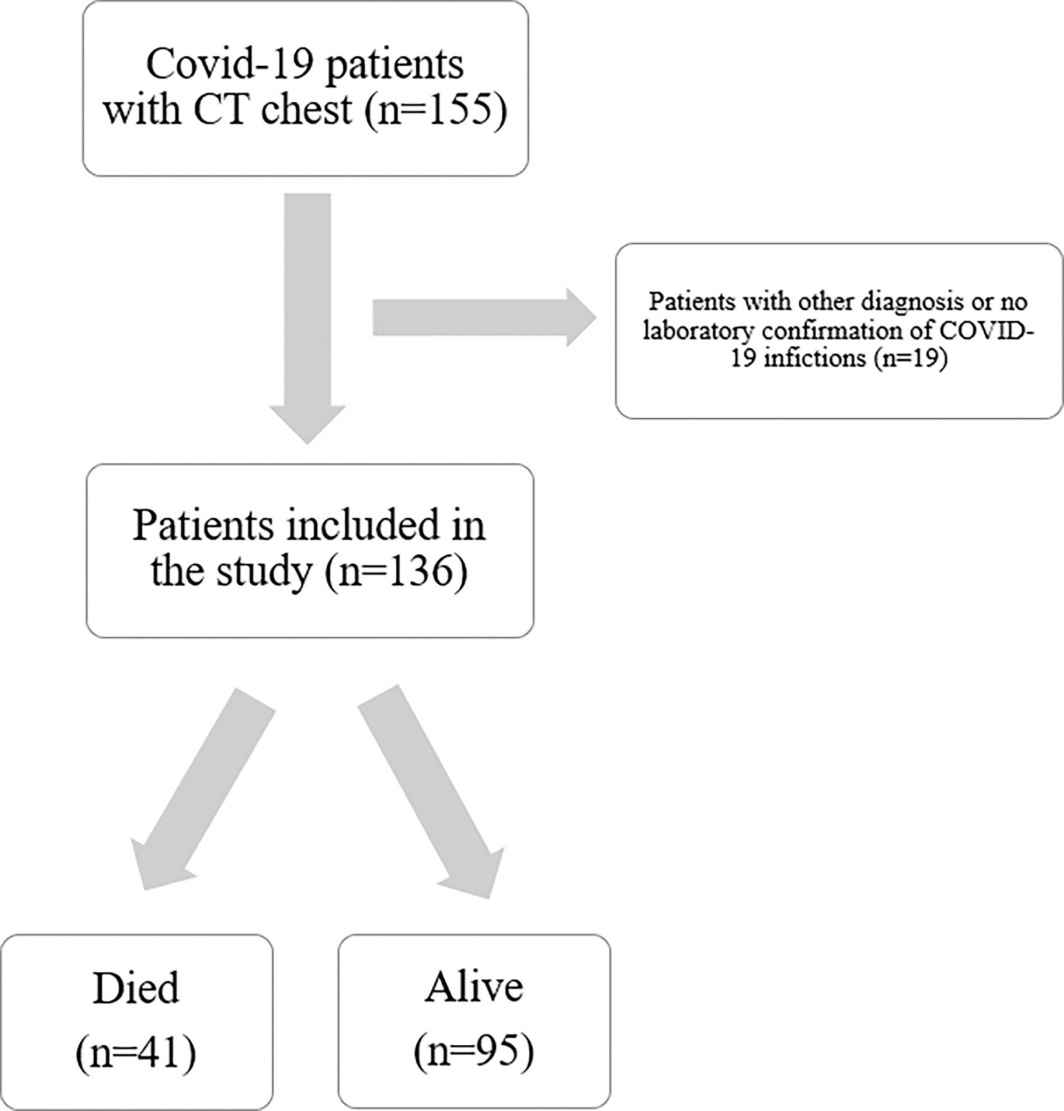
The flow chart of the inclusion and exclusion criteria.

This study was carried out at the King Abdullah Medical Complex in Jeddah, Saudi Arabia, one of the largest COVID-19 centers in the western province. The study included all adult COVID-19 patients who had a CT chest between January 2020 and April 2022. Patients under the age of 18 and those who had no reverse transcriptase polymerase chain reaction (RT-PCR), which is the gold standard for diagnosing COVID-19 infection, were excluded. The current study included a total of 136 patients. The flow chart of the inclusion and exclusion criteria is shown in (Fig 1).

### Chest CT protocol

CT imaging was performed with the following scanners: a multi-detector HiSpeed-Dual CT scanner with 64 channels (GE Healthcare). For CT examinations, the following parameters were used: tube voltage, 120 kVp; 150 mAs; collimation, 0.625–5 mm; pitch, 0.9; and image reconstruction (slice thickness, 2.5 mm/interval, 2.5 mm). All scans were performed from the upper thoracic inlet to the inferior costophrenic angle. CT scans were performed with the patient supine and at full inspiration.

### Image evaluation

The CT chest of COVID-19 patients was evaluated by two radiologists, each with a 10-year of experience, who measured vertebral BMD and assigned pulmonary severity scores (PSS). Both researchers agreed on the same method of measurement.

The measurements of vertebral BMD were obtained on unenhanced CT in the axial section, bone window (window level: 300 and window width: 1600 Hounsfield units). The first lumber vertebra (L1) was chosen as the measurement site, and ROI was placed away from the venous plexus and bone cortices. If L1 has pathology, the thoracic 12th vertebra (T12) was chosen [18]. As in previous studies [19,20], the measurements were obtained in Hounsfield units (HU), with a value of< = 100 HU set for low density. The vertebral BMD was measured using the conversion formulas described by Andrea Toelly et al. for CT with I.V contrast [21].

This study’s pneumonia severity score (PSS) was computed, as in previous studies [22,23]. The PSS was calculated using CT chest images, with each lobe calculated separately and the sum of the scores from the five lobes yielding the PSS. A score from 0–4 is assigned to each lobe. The PSS ranges from 0 to 20.

### Clinical examination

The patients’ age, gender, nationality, and comorbidities, including diabetes, hypertension, coronary artery disease, renal disease, chronic lung disease, and obesity, were obtained from the electronic medical records. The data on hospital stays, intensive care unit (ICU) admissions, and mortalities were also collected.

### Statistical analysis

Statistical analysis was performed using SPSS version 25.0 (IBM). Descriptive statistical analysis was used to describe patients’ demographics, clinical characteristics and radiological findings. The Chi-Square Test was used to compare categorical variables in the present study. Categorical variables were presented as numbers (n) and percentages (%). Continuous variables were presented as mean ± standard deviation values.

## Results

### Clinical and demographic characteristics

The current study is comprised of 136 COVID-19 pneumonia patients in total who met the qualifying requirements. (Table 1) provides a summary of each patient’s characteristics. The average patient age was 56.4 years, and the majority (73.5%) of patients were men. Diabetes (n = 66, 48.5%), hypertension (n = 56, 41.2%), and coronary artery disease (n = 17, 12.5%) were the most prevalent comorbidities. Among the 136 patients, 114 (83.8%) were hospitalized, of which approximately two-thirds required ICU admission (64%), and one-third succumbed to their deaths (30%). The average length of stay in the hospital was 28.4 days (Table 1).

**Table 1:** Demographic and clinical characteristics.

Variables	All patients (n = 136)
Age (years), mean ±SD	56.4± 14.3
Gender, n (%)	
Male	100 (73.5%)
Female	36 (26.5%)
**Comorbidities, n (%)**	
Diabetes	66 (48.5%)
Coronary Artery Disease	17 (12.5%)
Renal disease	7 (5.1%)
Chronic lung disease	9 (6.6%)
Obesity	9 (6.6%)
Hypertension	56 (41.2%)
**Clinical classification, n (%)**	
Mild	22 (16.2%)
Moderate/severe	114 (83.8%)
**Clinical outcomes**	
Hospital stays (days), mean +/- SD	28.4 (+/- 26.4)
Hospital admission, n (%)	114 (83.8%)
Intensive care unit admission, n (%)	87 (64%)
Mortality, n (%)	41 (30.1%)
**Radiological characteristics**	
Pneumonia Severity Score (PSS), n (%)	10.6 (26.5%)
BMD, n (%) < = 100 >100	12 (8.8%) 124 (91.2%)

### Radiological characteristics

The mean CT pneumonia severity score (PSS) was 10.6 at the time of admission. Patients with lower vertebral BMD (< = 100) numbered 12 (8.8%), while those with higher vertebral BMD (>100) numbered 124 (91.2%) (Table 1).

(Table 2) demonstrates that the group with low BMD is older than those with higher BMD (P<0.01). (Table 2) displays the correlation between vertebral BMD and other variables. Only 46 out of the total survived patients (n = 95) were admitted to the ICU, compared to all of the deceased (P<0.01). (Table 3) displays other comparable characteristics. The pneumonia severity score (PSS) at the time of admission can be used to predict whether a patient would survive a COVID infection, according to the findings of the logistic regression analysis (Table 4). An increased PSS at the time of admission, indicates a lower chance of survival. Age, gender, and BMD did not predict survival chances.

**Table 2:** Comparison of the patients with lower (< = 100 HU) and higher (>100 HU) vertebral BMD.

Variables	< = 100	>100	Test Statistic
	(n = 12)	(n = 124)	
Age in years, mean	**70.0**	**57.0**	F_1,134_ = 20.04, P<0.01^3^
Gender: Male	0.5	0.8	Χ21 = 3.74, P = 0.05^2^
Hypertension	0.8	0.4	Χ21 = 6.22, P = 0.01^2^
Diabetes	0.8	0.5	Χ21 = 3.69, P = 0.05^2^
Coronary Artery Disease	0.2	0.1	Χ21 = 1.88, P = 0.17^2^
Renal disease	0.1	0.0	Χ21 = 0.27, P = 0.60^2^
Chronic lung disease	0.1	0.1	Χ21 = 0.06, P = 0.80^2^
Obesity	0.1	0.1	Χ21 = 0.06, P = 0.80^2^
Hospital stays in days, mean	**21.0**	**20.0**	F_1,112_ = 0.00, P = 0.95^3^
ICU admission	0.7	0.6	Χ21 = 0.04, P = 0.84^2^
Alive	0.8	0.7	Χ21 = 0.17, P = 0.68^2^

**Table 3:** Comparison of the discharged patients and those that developed in-hospital mortality.

Variables	In-hospital mortality (n = 41)	Discharged (n = 95)	Test Statistic
Age in years, mean	**62.0**	**57.0**	F_1,134_ = 3.45, P = 0.07^3^
Gender: Male	0.8	0.7	Χ21 = 2.66, P = 0.10^2^
Hypertension	0.4	0.4	Χ21 = 0.00, P = 0.96^2^
Diabetes	0.6	0.4	Χ21 = 3.64, P = 0.06^2^
Coronary Artery Disease	0.1	0.1	Χ21 = 0.00, P = 0.94^2^
Renal disease	0.1	0.0	Χ21 = 0.57, P = 0.45^2^
Chronic lung disease	0.0	0.1	Χ21 = 0.29, P = 0.59^2^
Obesity	0.0	0.1	Χ21 = 0.29, P = 0.59^2^
Hospital stays in days, mean	**22.0**	**18.0**	F_1,112_ = 1.20, P = 0.28^3^
ICU admission	1.0	0.5	Χ21 = 33.06, P<0.01^2^
BMD: >100	0.9	0.9	Χ21 = 0.17, P = 0.68^2^

^1^Kruskal-Wallis.

^2^Pearson.

^3^Wilcoxon.

**Table 4:** Binominal logistic regression analysis of the data found significant in the univariate analysis for the prediction of mortality in patients with COVID-19.

Predictor	Estimate	SE	Z	p	Odds ratio	95% Confidence Interval	
						Lower	Upper
Intercept	7.66	2.15	3.55	< .001	2129	31.15	145548.74
Age	-0.03	0.02	-1.60	0.110	0.97	0.94	1.01
Admission CTC score	-0.37	0.09	-4.17	< .001	0.68	0.58	0.82
Gender							
M–F	-0.48	0.62	-0.77	0.438	0.62	0.18	2.08
BMD							
>100 –< = 100	-0.86	0.85	-1.01	0.315	0.42	0.08	2.26

Note. Estimates represent the log odds of "Alive = Yes" vs. "Alive = No"

## Discussion

The literature search revealed that this is the first study in Saudi Arabia and the Gulf region and the second in the Middle East to assess the significance of quantitative vertebral BMD and PSS acquired from chest CT and the link with death, which is a marker of the poorest clinical outcome in COVID -19.

Previous research found that the vast majority of COVID-19 cases were mild (81%). However, 14% of the cases were classified as severe, and 5% as critical [11,24,25]. In contrast to prior findings, only a small percentage of patients in the current study were classified as mild (16.2%). This is to be expected, given that most of the patients in the current study were hospitalized, resulting in a more moderate or severe disease classification (83.8%). Similar to previous studies, our study found severe outcomes such as increased hospital stays, ICU admission, or death.

The present investigation utilized ROI measurements of the L1 vertebral body and, if not available, T12, using a cutoff of 100 HU [18]. Many factors and comorbidities were found to affect the outcome of COVID-19, and BMD correlates with some of these factors [26]. Hounsfield of the vertebral body values was found to have a strong correlation with the T-score, and it is a meaningful tool for BMD screening [27–29].

Contrary to the current study, Tahtabishi et al. found that lower vertebral BMD is a significant and independent predictor of COVID-19 mortality. Their study included 63 patients with low BMD, with a mean age of 72.1 ± 12.6 years [30]. In their multicenter study, Kottlors et al. discovered that low BMD in COVID-19 patients was a risk factor for ICU admission. However, after accounting for gender and age, BMD was found to be a non-significant predictor [11]. In their study, the average age was 59.3 ± 16.2 years. Batistti et al. found that vertebral fractures have no independent effect on mortality in COVID-19 patients [31]. A recent study utilized a different method where total body BMD was calculated from MR images, and the study population was divided into five age groups, demonstrating that the correlation between low BMD and severity of COVID-19 is only significant in patients over 60 years of age [17]. Although age and BMD have a strong association in our patient population, we found no significant correlation between low BMD and the severity of COVID-19.

Many studies have shown a significant correlation between PSS and clinical disease score in COVID-19 patients, with excellent interobserver agreement [22]. Similarly, PSS was found to predict mortality, the significant factor in the present study. Like our findings several studies have found that the male gender and comorbidities such as hypertension and diabetes were highly prevalent [21,32].

Our study highlights the prognostic value of CT chest in COVID-19 patients, where such patient stratification is extremely useful in the current pandemic context as well as in any healthcare setting with limited resources. The PSS is a straightforward metric derived from CT chest that effectively predicts the outcome in COVID-19 patients. The current study’s strength stems from the fact that a number of COVID-19 cases, both hospitalized and outpatient, were assessed using chest CT.

There are a few limitations to the current investigations. First, the study used a single-center, retrospective design. Second, the sample size was not large, and all patients came from the same area. Third, in the current study, the majority but not all were hospitalized individuals; hence the findings cannot be generalized. Fourth, the patients did not receive dual energy X-ray absorptiometry (DEXA), the gold standard for detecting BMD.

## Conclusion

The current study found that BMD did not produce a prognostic advantage, and the PSS was the significant factor that could have predicted the outcome.

## Supporting information

S1 File (XLSX)
